# Free Sugar Content in Pre-Packaged Products: Does Voluntary Product Reformulation Work in Practice?

**DOI:** 10.3390/nu11112577

**Published:** 2019-10-25

**Authors:** Nina Zupanič, Maša Hribar, Nataša Fidler Mis, Igor Pravst

**Affiliations:** 1 Nutrition Institute, Tržaška cesta 40, SI-1000 Ljubljana, Slovenia; nina.zupanic@nutris.org (N.Z.); masa.hribar@nutris.org (M.H.); 2 Department of Gastroenterology, Hepatology and Nutrition, University Children’s Hospital, University Medical Centre Ljubljana, Bohoričeva 20, SI-1000 Ljubljana, Slovenia; natasa.fidler@kclj.si

**Keywords:** free sugar, ultra-processed food, food composition, sales data, nutrition label, Slovenia

## Abstract

Ultra-processed, pre-packaged foods are becoming a growing part of our diet, while displacing whole and minimally processed foods. This results in an increased intake of free sugar, salt, and saturated fats, that have a profoundly negative effect on health. We aimed to assess the trend in free sugar content in pre-packaged foods in Slovenia and evaluate the efficacy of industry self-regulations designed to combat the excess consumption of free sugar. A nation-wide data collection of the Slovenian food supply was performed in 2015 and repeated in 2017. In 2017, 54.5% of all products (*n* = 21,115) contained free sugars (median: 0.26 g free sugar/100 g). Soft drinks became the main free sugar source among pre-packaged goods (28% of all free sugar sold on the market) in place of chocolates and sweets, of which relative share decreased by 4.4%. In the categories with the highest free sugar share, market-leading brands were often sweeter than the average free sugar value of the category. This indicates that changes in on-shelf availability towards a greater number of healthier, less sweet products are not necessarily reflected in healthier consumers’ choices. Relying solely on voluntary industrial commitments to reduce free sugar consumption will likely not be sufficient to considerably improve public health. While some further improvements might be expected over the longer term, voluntarily commitments are more successful in increasing the availability of healthier alternatives, rather than improving the nutritional composition of the market-leading products. Additional activities are, therefore, needed to stimulate reformulation of the existing market-leading foods and drinks, and to stimulate the consumption of healthier alternatives.

## 1. Introduction

In the developed as well as in the developing countries, ultra-processed pre-packaged foods are representing an ever-greater share of our modern diet [1]. According to the NOVA food classification system, ultra-processed foods are inventions of modern food technologies, which contain very little or no whole foods and require little or no additional preparation before consumption [2]. Their main ingredients are usually isolated sugars, starches, oils, and fats, spiced with a combination of food additives to achieve desirable properties. Categories with the highest proportion of ultra-processed products include chocolates and sweets, jellies, ice-creams, and edible ices, biscuits, cakes, muffins and pastry, breakfast cereals, electrolyte drinks, meal replacements, soft drinks, ready meals, etc. [3,4]. 

From 2000 to 2013, the average increase in sales of ultra-processed goods among eighty different countries around the globe was 43.7%. In 2018, a cross-sectional study conducted by Monteiro and colleagues [5] reported that the average household availability of ultra-processed foods in Europe differed greatly among different countries, ranging from 10.2% in Portugal to 50.4% in the UK. In Canadians, 45% of daily caloric intake was met with the consumption of ultra-processed food products [6]. The highest reported proportion of ultra-processed foods in the diet was in the US, with an average of 57.9% of the daily energy intake [7]. 

Pre-packaged foods, especially ultra-processed foods are nutritionally imbalanced, higher in free sugar, total and saturated fat, salt, and lower in fiber and micronutrient content than fresh or home-cooked meals prepared from whole foods or minimally processed ingredients. Therefore, it comes as no surprise that the dietary share of ultra-processed foods is a strong predictor of poor diet quality [7,8,9,10]. In the US, the intake of ultra-processed foods contributes 90% of all added sugar consumed [7]. Moreover, the high palatability of those products frequently leads to overconsumption. Research showed that a percentage point increase in the household availability of ultra-processed foods results in a 0.25% increase in obesity prevalence [5]. 

High free sugar consumption remains one of the major health issues related to the modern diet, dominated increasingly by ultra-processed products. Defined by the World Health Organization (WHO), free sugars are mono- and disaccharides added to foods by manufacturers, cooks, or consumers, as well as sugars naturally present in syrups, honey, fruit juices, and fruit juice concentrates [11]. Our previous research, conducted in 2015, revealed that more than half of all pre-packaged products on the Slovenian market contained one or more forms of free sugar, listed under several different names. Moreover, it also identified food categories that contribute to free sugar consumption the most: Chocolate and sweets, soft drinks, biscuits, fruit and vegetable juices, and cereal bars [12]. Especially worrisome was high free sugar content in food groups, where consumers might not be aware of its presence and are generally perceived as healthy, such as baby foods, breakfast cereals, fruit yogurts, and cereal bars. High free sugar content in such pre-packaged goods may further hamper the adherence to the WHO [11], the European Society for Pediatric Gastroenterology Hepatology and Nutrition (ESPGHAN) [13], and the American Heart Association (AHA) recommendations [14], all of which advocate the limit of free sugar consumption to less than 10 or even 5% of the total daily energy intake and complete avoidance of free sugar for kids up to two years of age. To help the consumers meet the recommendations, pre-packaged products should be reformulated into healthier alternatives, while different public-health oriented legislative and educational actions must urge people to consume whole foods and minimally-processed products more often. 

In line with the WHO recommendations [11], Slovenian dietary guidelines now advise eating less than 10% of the daily caloric load in the form of free sugars [15]. Thus, among other health-related goals, the Slovenian Ministry of Health has set the target to lower free sugar consumption by decreasing the amount of sugar in pre-packaged products. While the attempt for taxation of soft drinks in 2015 was not successful due to strong opposition of the soft drink industry, the government agreed on voluntary industrial commitments to reduce free sugar content. To monitor the progress in practice, the present study aimed to evaluate the trends in total and free sugar content in pre-packaged goods, with the emphasis on processed and ultra-processed foods on the Slovenian market between 2015 and 2017. 

## 2. Material and Methods

### 2.1. Data Collection 2015 and 2017

Cross-sectional data collection in 2015 was carried out as described elsewhere [12]. In 2017, data collection was extended to five (previously three) major grocery chains (Spar, Mercator, Hofer, Lidl, and Tuš), with the network of shops widely accessible across the entire country. Sampling took place between February and June 2017 in major outlets in Ljubljana, Slovenia. All available products with the unique European/International Article Number (EAN) barcode were scanned with a mobile phone application (CLAS mobile phone application, developed specifically for this purpose by our institute), which enabled accelerated data collection and prevented duplicate entries. All scans were transferred into an online Composition and Labelling Information System (CLAS) database [16]. Additionally, each product was systematically photographed to facilitate the collection of all available information including nutritional composition, ingredients list, as well as nutritional and health claims copied into the database. Altogether, data of 21,115 pre-packaged products were scanned and photographed, excluding alcoholic beverages and dietary supplements. Unpackaged foods, such as loose fruits, vegetables, and nuts, as well as deli meats and cheese were not included in the present study. 

Upon re-checking all the entries, each item was categorized into one of the previously defined 49 food categories, as proposed by the Global Food Monitoring Initiative [17] and utilized in our previous study [12]. Each product was assigned into one of the following categories: Baby foods, biscuits, bread, breakfast cereals, butter and margarine, cakes, muffins and pastry, canned fish and seafood, cereal bars, cheese, chewing gum, chilled fish, chocolate and sweets, coffee and tea, cooking oil, cordials, couscous, cream, crisps and snacks, desserts, eggs, electrolyte drinks, frozen fish, fruits, fruit and vegetable juices, honey and syrups, ice cream and edible ices, jam and spreads, jelly, maize (corn), mayonnaise/dressings, meal replacements, meat alternatives, milk, noodles, nuts and seeds, other, other salt, pasta, pizza, pre-prepared salad and sandwiches, processed meat and derivatives, ready males, rice, sauces, soft drinks, soups, spreads, unprocessed cereals, vegetables, waters, and yogurt products.

The 49 food categories used in our previous study [12] remained the same, with the exception of unprocessed cereals, where flour was added, and coffee and tea category, into which pure coffee and tea were included additionally.

### 2.2. Calculation of Total and Free Sugar Content

**Figure 1:**
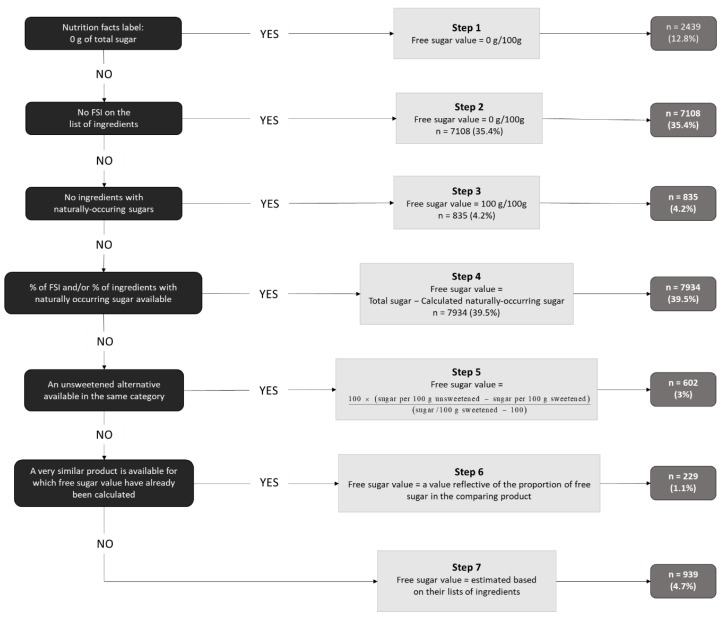
The decision tree algorithm used to estimate the free sugar content of packaged foods. The method was a modified version of the algorithm developed by Bernstein and colleagues [18] and has been implemented in our previous database analyses [12]. *n (%)* indicates the number and proportion of products included at each step.

The methodology and data analyses in the present paper have already been utilized in the previous total and free sugar assessment in 2015 [12]. Briefly, the ingredient list of every product was inspected for any form of free sugar, based on the WHO definition of free sugars [11]. Based on the information on total sugar content, which represents a mandatory part of every nutritional declaration in EU since 2017 and identified forms of free sugar in ingredient lists, free sugar content was calculated or estimated using an adapted step-by-step method initially developed by Bernstein and colleagues [18] and adapted accordingly in our previous study [12]. A seven-step algorithm is explained in detail in Figure 1. 

We determined the presence of free sugar in 20,949 products out of 21,115 entries in the original database and further calculated the exact free sugar content for 20,086 products. The remaining items for which free sugar content could not be assessed accurately enough due to too many missing information were excluded from further analyses. 

### 2.3. Sales-Weighted Total and Free Sugar Content

The database was further extended with the 12-month sales data provided by the two largest retailers in Slovenia, covering the majority of the national market. The sales data were nation-wide and contained information on EAN code, name of the product, quantity of food or beverage per packaging (kg or L), and the number of products sold. For 2015 analysis we obtained sales data of 8,620 products. In 2017, with some additional food groups included and slightly longer data collection time, sales data of 13,841 were matched with entries in the CLAS database. The sales-weighted average was calculated separately for each category resulting in a sales-adjusted total and free sugar content mean value. 

### 2.4. Share in Free Sugar Sales

To evaluate the relative contribution of each food category towards the overall free sugar consumption, shares in free sugar sales were calculated for each category. The final values are represented as ratios between the total sum of all free sugar sold in a certain category and the total sum of free sugar (kg) sold on the market in 2016 as a whole.

### 2.5. 2015–2017 Comparison between On-Shelf Availability and Sales Depending on Free Sugar Content

For the direct comparison of the total and free sugar content, we focused on five categories of particular interest, all comprising of an identical subset of products for both years. To make direct comparison possible, only products available in the initial three supermarket chains were included from both datasets. The on-shelf availability and relative importance in sales were calculated for each subset of products depending on their free sugar content within the category of interest. The items were stratified into groups of 1–2 g of difference in free sugar content. 

### 2.6. Data Accuracy 

The accuracy of the data collection and coding was assured using a confirmation procedure. The data collection was performed from the pictures of food labels taken and transcribed by students and re-checked by one of our researchers. All discrepancies were promptly resolved within the research team to ensure further data collection and coding consistency.

### 2.7. Statistical Analysis

Data management and processing was performed using the computer programs Microsoft SQL Server Management Studio V13.0, Microsoft Analysis Services Client Tools 13.0, Microsoft Data Access Components (MDAC) 10.0, Microsoft Excel 2016 (Redmond, WA, USA), and the program tool CLAS V1.0 (Composition and Labeling Information System, Nutrition Institute, Ljubljana, Slovenia). The data from the CLAS database was exported in the form of Microsoft Excel spreadsheets. 

Total and free sugar content in pre-packaged foods was presented as mean values, standard deviation (SD), and quartiles (min, 25th, 50th, 75th, max). The sales-weighted average for total and free sugar content was presented as an exact value without SD. The shares in free sugar sales were presented in percentages (%) of all free sugar sold on the market. The data for the comparison between on-shelf availability and sales were stratified by free sugar content and presented as the number of products available in each group. The sales factor for each group was added to the same figure to compare free sugar content in supply and demand in the Slovenian food supply. 

## 3. Results

### 3.1. A Median Total and Free Sugar Content in Different Food Categories 2017

Out of 20,949 evaluated products, 11,425 (54.5%) contained free sugar, while 9,524 (45.5%) did not. The exact free sugar content was calculated on a subset of 20,068 products, that made up a final database for the analysis. A median total sugar across all food categories was 4.5 g/100 g while median free sugar was 0.26 g/100 g. The median total and free sugar, mean total and free sugar content, as well as free-sugar as a proportion of total sugar per category are shown, in Table 1.

### 3.2. Trends in Sales-Adjusted Mean Free Sugar Content in Different Food Categories between 2015 and 2017

Using a combination of 12-month sales data provided by the retailers and detailed information on total and free sugar content, we were able to calculate sales-adjusted mean free sugar content for each of 49 food categories (Supplementary Table S1). The sales-adjusted mean in 2017 was higher than regular mean in the following categories: Biscuits (+18.8%), bread (+10.8%), breakfast cereals (+30.1%), cereal bars (+5%), chocolate and sweets (+1.5%), desserts (+5.2%), fruit and vegetable juices (+4.5%), honey and syrups (+9.1%), jam and spreads (+17.8%), pizza (+7.3%), soft drinks (+13.2%), and spreads (+50.6%). The higher sales-adjusted mean was observed in all categories with a notable contribution in overall free sugar intake, such as soft drinks, chocolate and sweets, and biscuits. On the contrary, considerably reduced sales-weighted free sugar mean was observed in cakes, muffins, and pastry, electrolyte drinks, pre-prepared salads and sandwiches, ready meals, and yogurts. 

**Figure 2:**
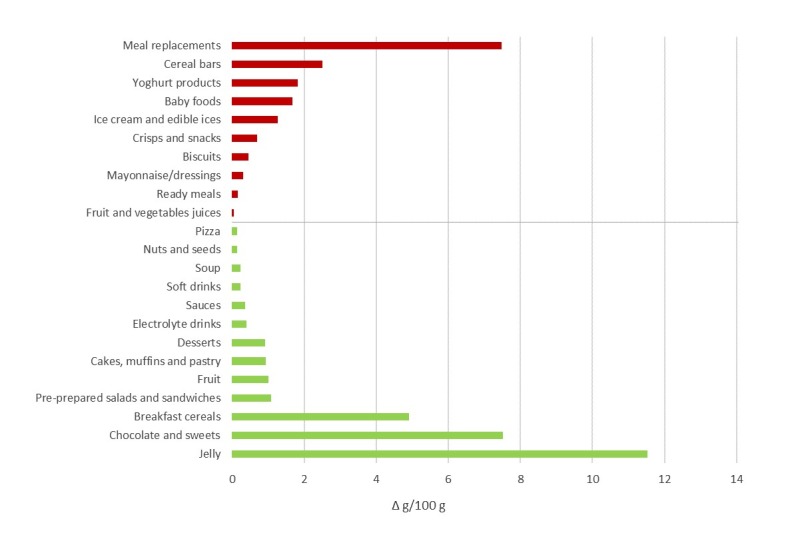
Trends in sales-weighted mean free sugar content (g/100 g or mL) in selected food categories between 2015 and 2017. The red bars indicate the increases in mean free sugar content in 2017 compared to 2015, while green show the decreases.

The sales-adjusted mean free sugar content of pre-packaged foods in 2017 was compared to the data collected in 2015. A direct comparison was made only for categories with total sugar content above 0 g/100 g/mL and with the identical sampling approach used in both data collections. The trends in sales-weighted mean free sugar content between 2015 and 2017 are presented in Figure 2. The largest decrease in mean free sugar content among sold pre-packaged products was observed among jelly, chocolate and sweets, and breakfast cereals. Meanwhile, meal replacements saw the largest increase in sales-weighted average free sugar content, followed by cereal bars, yoghurt products, and baby foods.

### 3.3. Share in Free Sugar Sales

**Figure 3:**
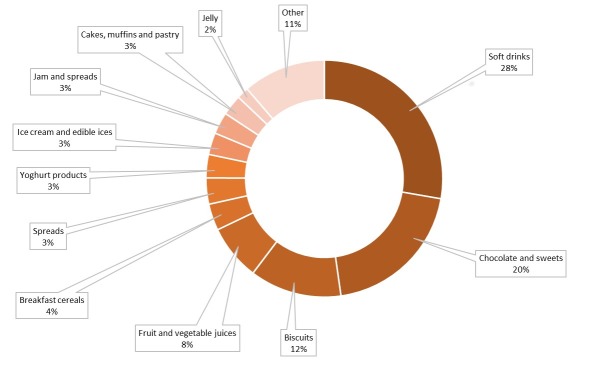
Relative contribution of different categories to the amount of free sugar (2017).

The relative importance of different food categories in free sugar consumption was assessed through sales data and previously calculated free sugar content. Figure 3 shows the relative proportion of free sugar sold per food category. The results revealed that with nearly 30% of all free sugar sold in the Slovenian grocery stores soft drinks are the major contributor to the dietary free sugar on the market. Chocolate and sweets contributed another 20%, followed by biscuits (12%), fruit and vegetable juices (8%), and breakfast cereals (4%). Spreads, yoghurt products, ice creams, and edible ices, jam and spreads, as well as cakes, muffins and pastry each contributed another 3%. Jellies were responsible for 2% of all free sugar sold, while the remaining categories with less than 2%-share were combined under the category of “Other”.

### 3.4. On-Shelf Availability and Sales of Products with Different Free Sugar Content in 2015 and 2017

To explore the trends in availability and sales of products across the entire range of free sugar content within each food category, five specific categories were chosen for comparison between years 2015 and 2017. The following categories of interest were selected based on their importance in overall free sugar consumption and The Slovenian Resolution on Nutrition and Physical Activity for Health (SRNPAH) 2015–2025 priorities: Yoghurts, biscuits, breakfast cereals, cakes, muffins, and pastry, and soft drinks [15]. 

**Figure 4:**
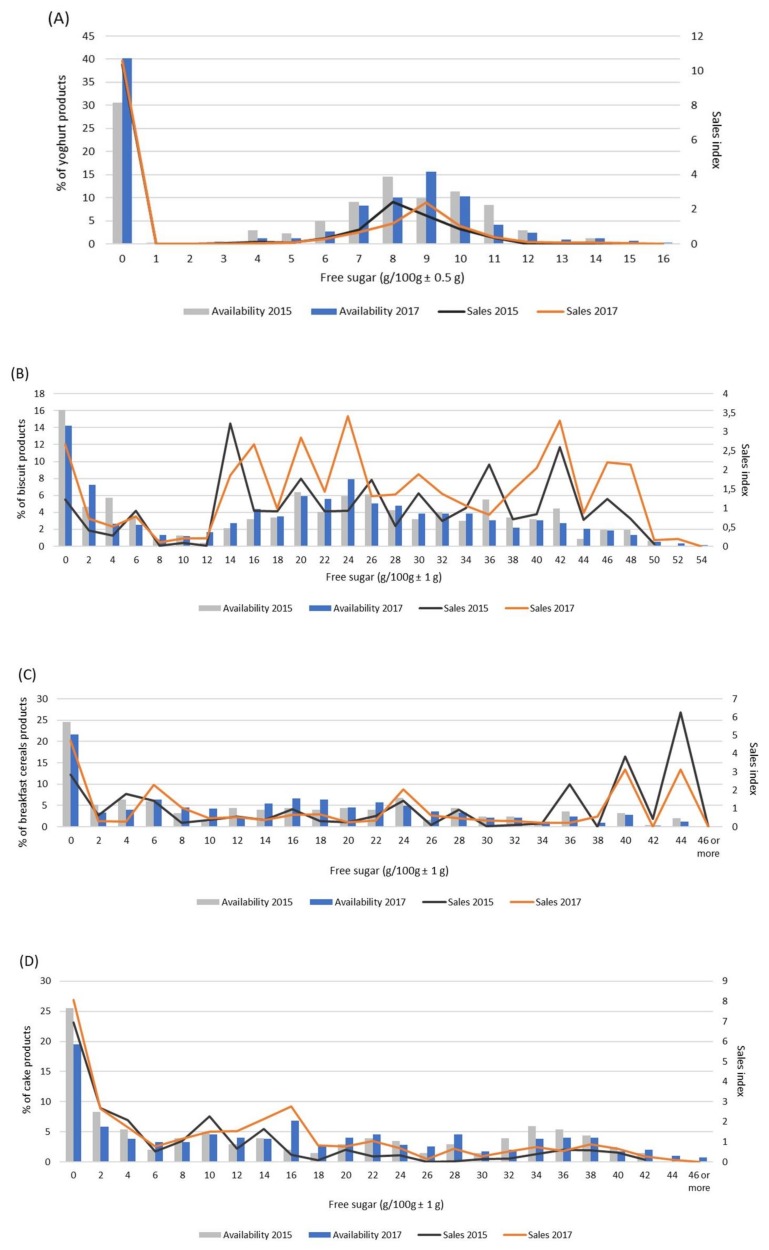

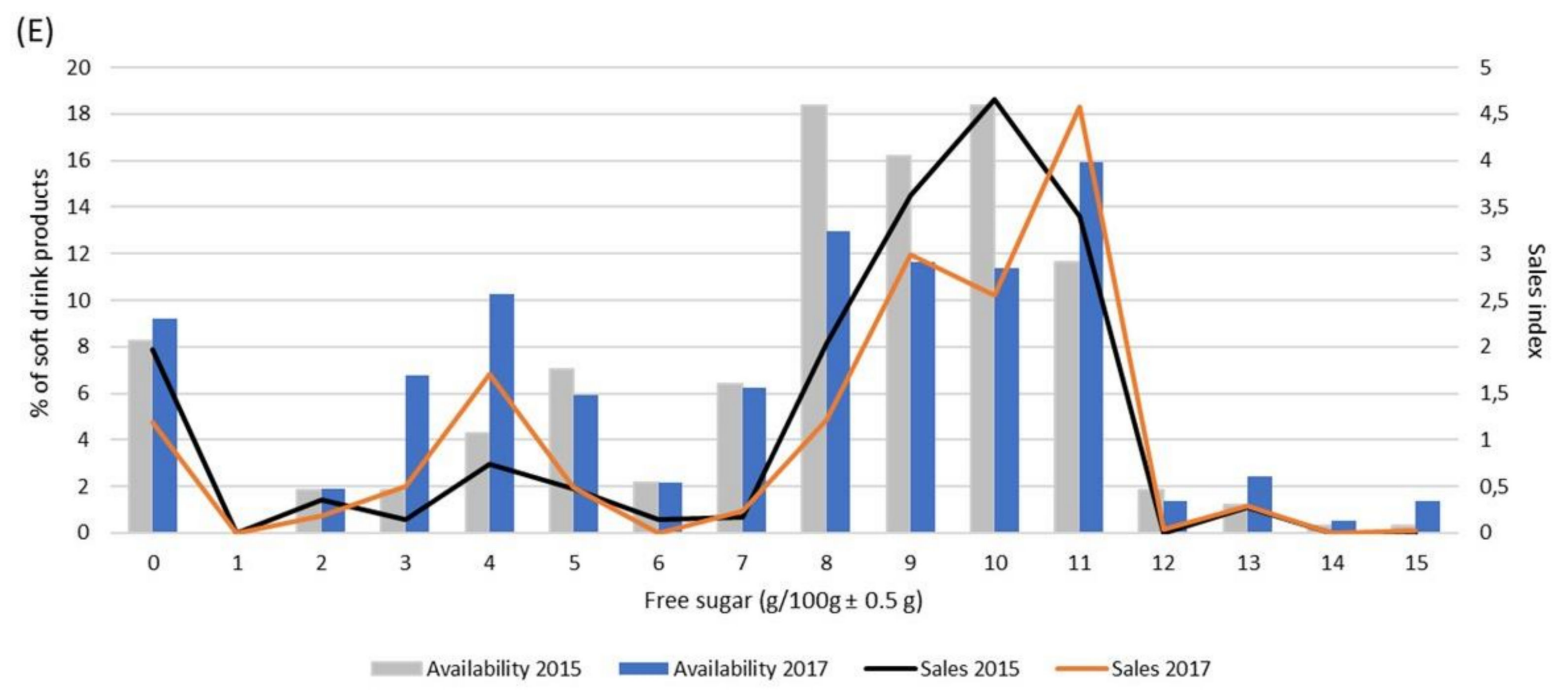
The comparison between the availability and sales of food products in relation to the free sugar content in 2015 and 2017. (**A**) Yogurts, (**B**) biscuits, (**C**) breakfast cereals, (**D**) cakes, muffins and pastry, and (**E**) soft drinks.

Altogether, the between-year comparison revealed similar general distribution patterns in availability as well as in sales, but also showed some interesting trends in consumers’ preferences and buying choices. In the category of yoghurts (Figure 4A), the availability of plain yoghurt varieties increased from 30% to 40% of all products available. However, the amount of plain yoghurt sold remained unchanged despite the increased availability on the market. On the other hand, free sugar content in fruit yoghurts available on the market increased. The highest proportion of fruit yoghurts on the Slovenian market in 2017 contained 9 g compared to 8 g/100 g in 2015. The sales trend mirrored the conditions on the market, meaning that people consuming fruit yogurts in 2017 consumed higher amounts of free sugar than in 2015.

Favorable trends were observed in breakfast cereals sales, with the increased demand for unsweetened minimally processed varieties. Concomitantly, the sales of the products with the highest free sugar content nearly halved, but the overall sales of very sweet breakfast cereal varieties remained high (Figure 4C). 

The two categories: Biscuits (Figure 4B) and cakes, muffins, and pastry (Figure 4D) contained several different types of snacks and desserts and are therefore more divers regarding free sugar content. While unsweetened varieties became a more common choice among consumers, biscuits with the highest contents of free sugar also gained popularity. Free sugar content in the most often bought cakes, muffins, and pastry also increased from 10 g to 16 g/100 g. 

In soft drinks, which in 2017 contributed the highest proportion of free sugar towards the entire free sugar load sold on the market, there were some noticeable changes as well. From 2015 to 2017, the proportion of soft drinks with zero free sugar increased slightly, but their sales plummeted almost by half, now representing 10% of all soft drinks sold on the Slovenian market (data not shown). Instead, consumers were more likely to choose drinks marketed as flavored waters and other soft drinks with low free sugar content (from 3 to 5 g of free sugar/100 mL), assortment of which increased by two-fold. On the sweeter side of the shelf, the most popular drinks became those with 11 g of free sugar per 100 mL compared to 10 g of free sugar in 2015, following the in-store offer trend (Figure 4E).

## 4. Discussion

The current analysis is an extended follow-up analysis of the total and free sugar content of pre-packaged foods in Slovenia in 2015 [12]. In line with the previous findings, in 2017, the highest free sugar content was found in honey and syrups, jellies, jam and spreads, chocolates and sweets, cereal bars, and biscuits. In 2015, the major source of free sugar sold with pre-packaged foods were chocolates and sweets, accounting for 34% of all free sugar sold in Slovenian main grocery chains, followed by soft drinks with 24% of the share. In 2017, soft drinks became the main free sugar source among pre-packaged goods, now topping the ranking with 28%. While the share of chocolates and sweets decreased by 4.4%, the higher share of free sugar gets consumed with biscuits. 

Especially worrisome is the finding that in the categories with the highest free sugar share market-leading brands are often sweeter than the average free sugar value of the category. Moreover, our data showed that changes in on-shelf availability towards a greater number of healthier, less sweet products do not necessarily reflect in healthier consumers’ choices. From 2015 to 2017 the availability of flavored waters, which are generally less sweet than carbonated soft drinks and ice teas increased markedly. Given the broader market offer, there was also a proportional increase in sales of these less-sweet alternatives. However, the observed increase in sales did not occur on the account of sweeter soft drinks, but rather on the account of unsweetened or artificially sweetened soft drinks with zero free sugar, and possibly waters. On the sweeter side of the shelf, the most popular soft drinks got even sweeter. The sales now peak at 11 instead of 10 g of free sugar/100 mL. Thus, in two years-time the relative amount of free sugar consumed with soft drinks increased even further, now accounting for 28% instead of 24% of all free sugar sold on the Slovenian market. Similarly, despite the higher choice of plain and sugar-free yoghurts, in 2017 people were buying sweeter yoghurts than before. 

On the other hand, a handful of food categories with high free sugar content, such as chocolate and sweets, jellies, and breakfast cereals have undergone substantial improvement. More people are buying sugar-free breakfast cereals even though the sweetest choices on the market are still among the most popular consumers’ choices. However, the trend is promising, especially as breakfast cereals are regularly perceived as a healthy breakfast choice, but can contain up to 44 g of free sugar/100 g. Among the sweetest and most commonly bought brands of breakfast cereals are those heavily marketed to children [19,20], which further counteracts the efforts to reduce their demand and divert consumers towards healthier options. 

Given the increasing number of calories consumed in a form of pre-packaged ultra-processed products, it is thus of utmost importance to nutritionally reformulate those products into more health-favorable alternatives and, preferably, encourage people to consume unprocessed whole foods or minimally processed food products more often. To achieve this goal, different strategies have been proposed, although the results have yielded limited effects. Several food and beverage manufacturers around the world, including Slovenia, have pledged to reduce the amount of added or free sugar and other nutrients of concern, such as salt and saturated fats, but our findings, as well as findings of other researchers [21,22], support the premise that industry self-regulations are not sufficient to considerably improve diet quality. In Slovenia specifically, industry pledges have dissuaded advertising of food to children, encouraged front-of-pack labeling of energy content, and promoted the development of healthier alternatives. However, the pledges were not signed by all food companies (e.g., they do not apply for supermarket own-brands, which have a considerable market share) and were focused on a very few food categories, such as soft drinks and dairy products. Moreover, studies have shown that so-called “nutrients-to-limit” are often replaced with food additives or other highly-processed ingredients rather than with beneficial whole foods [23], and even when the new ingredient is a healthier alternative, the final product is generally only less unhealthy, although still unhealthy. Additionally, food reformulation is less likely to occur on niche products, while in the case of market-leading products food manufacturers do not want to risk migration of the consumers to other brands due to changes in the sensory properties of their products. Voluntary product reformulations thus often result in minor changes in nutrient profiles that have very little impact on the overall diet quality while simultaneously divert attention from more impactful public health actions [24].

The present study was performed using a very extensive database, covering the great majority of the Slovenian food market, which enabled a very precise assessment of the market situation. Even more accurate estimation of the amount of free sugar sold and relative importance of different categories and brands in free sugar consumption was possible with the use of the nationwide 12-month sales data provided by the retailers. The major limitation of the present study is its focus solely on pre-packaged products, which makes it impossible to assess the share unprocessed and minimally processed foods represent in the overall free sugar intake. Lack of this important information makes hard to grasp the extent of the problem that free sugar from pre-packaged products poses to our diets. Another limitation of the study presents the fact that data from nutrition labels were used instead of chemical analyses, but it should be emphasized that such an approach enabled the inclusion of an extremely large dataset of foods. Furthermore, there is also no experimental method available for accurate determination of free sugar content due to its chemical indistinguishability from naturally-occurring sugars. Lastly, we were not granted access to the sales data from all the retailers. However, the largest retailers with the majority of the market share in the country were included in the study. 

## 5. Conclusions

Pre-packaged products in Slovenia have undergone some minor improvements regarding the amount of free sugar in certain categories, but the overall free sugar content remains high. To cope with the problem more efficiently national public health authorities will need to design a comprehensive multi-pronged approach. Some countries have already introduced taxation on sugar-sweetened beverages and/or unhealthy foods, which has proven to be an effective part of the strategy for fighting obesity [25]. Another, and potentially less challenging, policy option would be to specify upper acceptable sugar levels in key food categories. Such thresholds would set a clear goal for the industry and enable the evaluation of the progress in reducing free sugar content in pre-packaged foods, while at the same time offer guidelines for schools’ and other public institutions’ food purchases. 

Relying solely on voluntary ‘public-private partnership’-agreed sugar reductions, which expect the private sector to work in the public interest will most likely gain very limited results. Thus, it is important that industry-proposed actions do not divert the focus from other more effective strategies in form of statutory regulations and health risk/benefit communication with consumers, which will improve the state of public health through nutrition and reduce the rate of obesity and chronic non-communicable diseases. 

## Figures and Tables

**Table 1:** Total and free sugar content (g/100 g or g/100 mL) of pre-packaged food products divided by food categories (2017 data, Slovenia). Mean values, SD, quartiles (min, 25th, 50th/median, 75th, max), and mean free sugar as a proportion of total sugar are shown for each category.

Food Category	*n*	Total Sugar (g/100 g or g/100 mL)						Free Sugar (g/100 g or g/100 mL)						Free Sugar (% of total sugar)
		Mean (SD)	Min	25th	50th	75th	Max	Mean (SD)	Min	25th	50th	75th	Max	
Baby foods	216	14.5 (17)	0	7.9	10	12.7	94	10.2 (17.2)	0	0	6.7	10.7	94	56.7
Biscuits	1302	24.0 (14.9)	0	12.7	25	35.2	77	22.2 (14.8)	0	8.8	23.4	33	75.1	82.8
Bread	224	3.4 (2.8)	0.2	1.6	2.6	4.1	19	2.1 (3)	0	0	2.6	3.3	6.6	61.9
Breakfast cereals	443	17.7 (11.3)	0	7.7	18.8	25	45	14.5 (11.8)	0	3.4	14.2	22.7	44.4	67.1
Butter and margarine	128	0.5 (0.6)	0	0	0.5	0.6	3.9	0 (0.1)	0	0	0	0	0.8	1.3
Cakes, muffins, and pastry	601	20.8 (13.1)	0.4	14	22.5	33.5	60	21.9 (12.1)	0	11.7	21	31.8	59.9	89.2
Canned fish and seafood	304	0.9 (1.5)	0	0	0	1.5	7.4	0.5 (1.1)	0	0	0	0	7.1	14.1
Cereal bars	68	29.6 (6.1)	1.8	27	30.9	33.4	40	27 (5.6)	1.2	23.1	28.3	31	35.5	91.2
Cheese	842	2.0 (3)	0	0.1	1	3	33	0.1 (1.4)	0	0	0	0	28	1.1
Chewing gum	96	4.2 (17.3)	0	0	0	0	86	4 (17.2)	0	0	0	0	86	5.2
Chilled fish	37	0.4 (0.7)	0	0	0.4	0.6	3.8	0.1 (0.6)	0	0	0	0	3.7	8.1
Chocolate and sweets	1853	50.8 (17.3)	0	43.1	51.1	59	100	49.5 (18.8)	0	39.4	48.0	57.1	100	93
Coffee and tea	1063	7.1 (17.9)	0	0	0	0	89	5.3 (15.9)	0	0	0	0	89	12
Cooking oils	420	0 (0)	0	0	0	0	0	0 (0)	0	0	0	0	0	0
Cordials	140	31.7 (15.6)	0	9.0	10.3	52.2	88	31.7 (15.6)	0	9.0	10.3	52.2	88	100
Couscous	21	2.06 (0.8)	0.8	1.7	2.2	2.5	4.1	0.1 (0.6)	0	0	0	0	2.5	4.4
Cream	161	4.5 (3.9)	0	3	3.4	4.2	39	1.5 (4.2)	0	0	0	0	39	15.5
Crisps and snacks	435	2.9 (3.5)	0	1	2	3.4	41.3	1.7 (2.9)	0	0	0.7	2.5	35.6	40.6
Desserts	277	13.9 (10.6)	0	9.8	13	15	74	10.3 (10)	0	6	9.7	12.1	73.4	63.6
Eggs	83	0 (0)	0	0	0	0	0	0 (0)	0	0	0	0	0	0
Electrolyte drinks	27	16.3 (25.5)	0	3.9	4.2	6	75	16.3 (25.5)	0	3.9	4.2	6	75	92.6
Frozen fish	116	0.7 (1)	0	0	0.5	1	4.2	0.3 (0.9)	0	0	0	0	4.1	16.5
Fruits	413	35.4 (24.4)	0	12.8	30.5	59	88	10.7 (19.6)	0	0	0	10.6	79.1	29.2
Fruit and vegetables juices	457	9 (3)	0	8	9.7	11	16	9.0 (3)	0	8	9.7	11	16	99.6
Honey and syrups	197	86.6 (15.3)	21	79.5	95	99	100	86.6 (15.3)	21	79.5	95	99	100	100
Ice cream and edible ices	395	24 (6.3)	10	21	24	26.8	76	21 (6.6)	0	18.5	21.8	24.1	61	87.4
Jam and spreads	333	48.1 (13.6)	2	38.2	50	58.3	82.2	43.2 (15.9)	0	32.9	45.2	54.5	79	87.4
Jelly	144	56.2 (9.7)	39	47.2	54	64	82	56.2 (9.7)	39	47.2	54	64	82	100
Maize (Corn)	5	2.6 (1.5)	0	1.4	2.5	3.95	5.4	0.9 (1.1)	0	0	0	2.3	2.3	36.5
Mayonnaise/dressings	104	4.1 (3.5)	0.2	1.5	2.7	6	17	3.7 (3.4)	0	1.3	2.4	5.3	17	87.3
Meal replacements	29	13.3 (7.7)	0.5	6.8	13.4	18.7	36.5	11.4 (7.7)	0	5.9	11.3	16.9	33.5	76.1
Meat alternatives	111	1.8 (2.9)	0	0.2	0.5	1.83	13	0.3 (0.8)	0	0	0	0	5.8	15.8
Milk	321	6.1 (6)	0	4.4	4.8	6.5	55	2.2 (5.6)	0	0	0	2.9	51	24.6
Noodles	147	2.4 (1.5)	0	1.2	2.8	3.6	6.2	0 (0)	0	0	0	0	0	0
Nuts and seeds	398	9.1 (13.1)	0	1.88	4.3	6.9	69	2.7 (8.2)	0	0	0	0	55.7	6.1
Pasta	635	2.7 (1.6)	0	1.4	3	3.7	13.8	0.1 (0.5)	0	0	0	0	4.1	2.2
Pizza	52	3.2 (1.2)	1.3	2.3	3.2	4.2	5.4	2.3 (1.2)	1.2	1.4	2.3	3.1	4.7	69.9
Pre-prepared salads and sandwiches	36	2.8 (1.9)	0	1.2	2.5	3.4	7.8	1.8 (1.5)	0	0.6	1.3	2.4	5.6	59.9
Processed meat and derivatives	1558	0.5 (0.5)	0	0.1	0.5	0.5	5.6	0.3 (0.5)	0	0	0.1	0.5	5.6	54
Ready meals	355	2.4 (2.3)	0	0.9	1.9	3.3	15.6	1 (1.8)	0	0	0.2	1.4	12.3	34.6
Rice	158	0 (0)	0	0	0	0	0	0 (0)	0	0	0	0	0	0
Sauces	687	10.3 (12.7)	0	2.5	5.3	11.6	65	7.5 (12.5)	0	0	2	7	64.8	47
Soft drinks	529	7.1 (4.2)	0	4.1	8	10	37	7.1 (4.2)	0	4.1	8	10	37	89.2
Soup	189	0.6 (0.7)	0	0.3	0.5	0.8	3.7	0.4 (0.5)	0	0	0.2	0.5	3.5	89.2
Spreads	496	10.2 (19)	0	0.5	1.1	5.5	64.3	8.3 (17.6)	0	0	0.2	2	58.5	41.6
Unprocessed cereals	399	1.6 (2.1)	0	0.7	1.2	2	26.3	0.2 (1.4)	0	0	0	0	24	2.7
Vegetables	997	3 (4.7)	0	0.4	1.6	3.9	49	0.5 (2.5)	0	0	0	0	36.1	10.1
Waters	132	0 (0)	0	0	0	0	0	0 (0)	0	0	0	0	0	0
Yoghurt products	722	9.8 (4.4)	2.1	4.7	11.05	13	21.5	6.1 (4.8)	0	0	7.6	9.7	18.5	48

## Supplementary Materials

The following are available online at https://www.mdpi.com/2072-6643/11/11/2577/s1. Supplementary Table S1: Mean total and mean free sugar content (in g per 100 g or mL) of pre-packaged food products in 2017 divided by food categories.
