# Supplementary material for: Free Sugar Content in Pre-Packaged Products: Does Voluntary Product Reformulation Work in Practice?
Source: Nutrients. 2019 Oct 25;11(11):2577. doi: 10.3390/nu11112577 (PMC6893660; doi:10.3390/nu11112577)
Supplement: Supplementary file 1 [file nutrients-11-02577-s001.pdf]

Zupanič et al. Free Sugar Content in Pre-Packaged Products: Does Voluntary Product Reformulation Work in Practice?

Supplementary Table S1: Mean total and mean free sugar content (in g per 100 g or mL) of pre-packaged food products in 2017 divided by food categories

	Full sample		Sample with available sales data			
	Mean sugar content		Mean sugar content		Sales-weighted sugar content	
**Food category**	**Mean total sugar**	**Mean free sugar**	**Mean total sugar**	**Mean free sugar**	**Total sugar**	**Free sugar**
Baby foods	14.5	10.2	15.6	11.3	13.2	9.6
Biscuits	24	22.2	22.6	20.8	28.7	27.4
Bread	3.4	2.1	3.5	2.3	3.7	2.3
Breakfast cereals	17.7	14.5	17.9	14.7	23.1	20.7
Butter and margarine	0.5	0	0.5	0	0.5	0
Cakes, muffins, and pastry	20.8	21.9	19.5	17.4	14.6	13.1
Canned fish and seafood	0.9	0.5	0.9	0.3	0.9	0.3
Cereal bars	29.6	27	30.0	26.9	31	28.4
Cheese	2	0.1	2.1	0.1	2.4	0
Chewing gum	4.2	4	5.7	5.5	0.5	0.4
Chilled fish	0.4	0.1	0.5	0.2	0.6	0.1
Chocolate and sweets	50.8	49.5	50.9	47.3	53.7	50.3
Coffee and tea	7.1	5.3	7.2	6.3	4.1	3.4
Cooking oils	0	0	0	0	0	0
Cordials	31.7	31.7	6.6	8.3	6.6	8.3
Couscous	2.1	0.1	2.0	0.2	0.5	0.0
Cream	4.5	1.5	4.4	1.6	4	0.9
Crisps and snacks	2.9	1.7	2.7	1.7	2.5	1.6
Desserts	13.9	10.3	14.1	11.8	14.4	10.9
Eggs	0	0	0	0	0	0
Electrolyte drinks	16.3	16.3	17.8	17.8	4.3	4.3
Frozen fish	0.7	0.3	0.9	0.5	0.7	0
Fruit	35.4	10.7	36.7	11.3	31.5	5.9
Fruit and vegetables juices	9	9	8.8	8.8	9.4	9.4
Honey and syrups	86.6	86.6	87.2	87.2	95.3	95.3
Ice cream and edible ices	24	21	24.8	22.3	23.4	20.6
Jam and spreads	48.1	43.2	47.9	44.1	54.2	52.5
Jelly	56.2	56.2	56.5	56.5	53.8	53.8
Maize (Corn)	2.6	0.9	2.6	0.9	2.5	0
Mayonnaise/dressings	4.1	3.7	3.7	3.5	2.2	1.9
Meal replacements	13.3	11.4	13.3	11.3	9.2	8.1
Meat alternatives	1.8	0.3	1.7	0.2	1.8	0.1
Milk	6.1	2.6	6.1	2.0	0.1	0.1
Noodles	2.4	0	2.4	0	2.6	0
Nuts and seeds	9.1	2.3	9.6	2.7	7.6	0.6
Other	25.4	18.9	28.0	21.1	35.6	32.3
Other—salt	0.7	0	0.4	0	0	0
Pasta	2.7	0	2.8	0.1	3.2	0
Pizza	3.2	2.3	2.8	2.1	3	2.5
Pre-prepared salads and sandwiches	2.8	1.8	1.3	0.7	2	0.9
Processed meat and derivatives	0.5	0.3	0.5	0.4	0.4	0.3
Ready meals	2.4	1	2.1	0.7	1.9	0.6
Rice	0	0	0	0	0	0
Sauces	10.3	7.5	11.0	8.5	8	5.5
Soft drinks	7.1	7.1	7.4	7.4	8.2	8.2
Soup	0.6	0.4	0.6	0.4	0.4	0.1
Spreads	10.2	8.3	9.6	8.1	18.9	16.7

	Full sample		Sample with available sales data			
	**Mean sugar content**		**Mean sugar content**		**Sales-weighted sugar content**	
Unprocessed cereals	1.6	0.2	1.7	0.2	1.2	0
Vegetables	3.00	0.50	3.1	0.6	2.4	0.3
Waters	0	0	0	0	0	0
Yoghurt products	9.8	6.1	9.1	5.4	7.3	3.3
